# Treatment Response Assessment Maps (TRAMs), a new tool for CNS lymphoma

**DOI:** 10.1002/jha2.346

**Published:** 2022-01-11

**Authors:** Thomas Millard, Ian Chau, Sunil Iyengar, Dima El‐Sharkawi, David Cunningham, Bhupinder Sharma

**Affiliations:** ^1^ Haemato‐oncology unit the Royal Marsden Hospital London UK; ^2^ The Institute of Cancer Research London UK


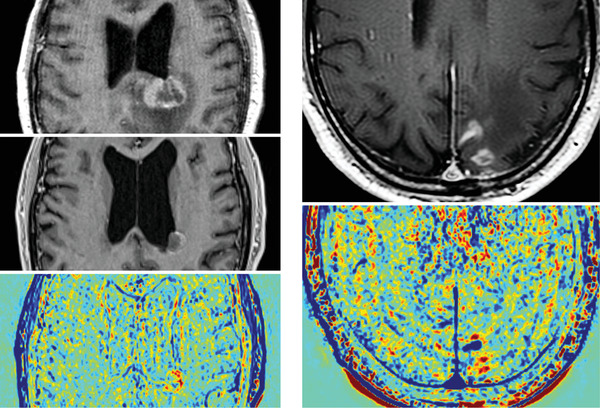


In the case of a 62‐year‐old man with biopsy confirmed primary diffuse large B‐cell lymphoma of the CNS treated with methotrexate, cytarabine, thiotepa and rituximab (MATRix) chemoimmunotherapy and busulphan/thiotepa autograft, follow‐up contrast enhanced magnetic resonance imaging (CE‐MRI) demonstrated ongoing peripheral enhancement at the site of original lesion (middle left). Here we show that treatment response assessment maps (TRAMs) can be used to differentiate active disease from benign enhancement.

Gadolinium enhancement is a feature of active lymphoma, but contrast enhancement is not specific as it is also seen following disruption of the blood brain barrier due to biopsy, inflammation and haemorrhage. With TRAMs, further magnetic resonance images are acquired approximately an hour post‐contrast administration and subtracted from the initial images providing a colour map. *‘Red’* regions have retained and *‘blue’* regions cleared contrast; contrast clearance is associated with active disease. TRAMs efficacy in discriminating viable lymphoma from post‐biopsy change at baseline is shown in another patient by the right sided images. In the case of this 67‐year‐old man, with biopsy proven primary CNS lymphoma, it is not possible to distinguish post‐biopsy change from possible remaining macroscopic lymphoma on the top right CE‐MRI. The bottom right TRAMs however discriminates the MRI findings into blue (viable lymphoma) and red (post‐biopsy benign) changes.

In the present case, the lesion (top left) had residual peripheral contrast enhancement following treatment (middle left). The red signal on TRAMs (bottom left) though indicates benign enhancement, enabling a decision not to proceed to radiotherapy. To the best of our knowledge the patient is still in complete remission nearly two years post‐imaging.

Early work has been published in some tumour types, however, to our knowledge this is the first report describing TRAMs as a new concept in CNS lymphoma. TRAMs has potential as a problem solving tool in CNS lymphoma as it allows MRI contrast enhancing regions to be stratified into areas of viable lymphoma and benign enhancement, increasing accuracy of the staging and response assessment used to guide patient management decisions.

